# Canine Distemper Virus in Wild Carnivore Populations from the Czech Republic (2012–2020): Occurrence, Geographical Distribution, and Phylogenetic Analysis

**DOI:** 10.3390/life12020289

**Published:** 2022-02-15

**Authors:** Eliška Kličková, Lenka Černíková, Aurélie Dumondin, Eva Bártová, Marie Budíková, Kamil Sedlák

**Affiliations:** 1 Department of Molecular Biology, State Veterinary Institute Prague, Sídlištní 136/24, Praha 6, 165 03 Lysolaje, Czech Republic; ela.vitaskova@seznam.cz (E.K.); lenka.cernikova@svupraha.cz (L.Č.); 2 Department of Biology and Wildlife Diseases, Faculty of Veterinary Hygiene and Ecology, University of Veterinary Sciences Brno, Palackého třída 1946/1, 612 42 Brno, Czech Republic; 3 Lycée Agro-Viticole, 84 Avenue du Général de Gaulle, CS 90113, CEDEX, 33295 Blanquefort, France; aurelie.dumondin@orange.fr; 4 Department of Mathematics and Statistics, Masaryk University, Kotlářská 267/2, 611 37 Brno, Czech Republic; budikova@math.muni.cz; 5 State Veterinary Institute Prague, Sídlištní 136/24, Praha 6, 165 03 Lysolaje, Czech Republic; kamil.sedlak@svupraha.cz

**Keywords:** badgers, foxes, hemagglutinin, martens, raccoons, RT-PCR, sequencing

## Abstract

Canine distemper is a highly contagious viral disease in carnivores and represents a serious threat for both wild and domestic animals. The aim of our study was to monitor the occurrence of the canine distemper virus in wildlife from the Czech Republic, reveal the H gene heterogeneity in positive samples and perform subsequent phylogenetic analysis. In total, 412 wild animals of 10 species were included in the study: 219 red foxes (*Vulpes vulpes*), 79 European badgers (*Meles meles*), 47 European otters (*Lutra lutra*), 40 stone martens (*Martes foina*), 10 pine martens (*M. martes*), 7 raccoons (*Procyon lotor*), 5 undetermined martens (*Martes* sp.), 2 wolves (*Canis lupus*), 1 European polecat (*Mustela putorius*), 1 free-ranging ferret (*Mustela putorius furo*), and 1 free-ranging American mink (*Neovison vison*). Most animals were found dead or were killed by hunters during hunting seasons in the years 2012–2020 and came from all 14 regions of the Czech Republic. In the animals that were hunted, symptoms such as apathy, loss of shyness or disorientation were reported. Canine distemper virus (CDV) was detected by real-time RT-PCR in the tissues of 74 (18%) of the animals, including 62 (28%) red foxes, 4 (10%) stone martens, 3 (43%) raccoons, 2 (20%) pine martens, 2 (2.5%) European badgers and 1 (20%) undetermined marten. There was a statistical difference in positivity among animal species (*p* < 0.0001), regions (*p* = 0.0057), and the years of sampling (*p* = 0.0005). To determine the genetic characteristics of circulating variants of CDV in wildlife, 23 of 74 CDV variants were partially sequenced. Phylogenetic analysis showed that 21 variants belonged to the European lineage and two strains belonged to the European-Wildlife lineage. This study provides the first comprehensive overview of the prevalence and spatial distribution of CDV in wildlife in the Czech Republic, including molecular phylogenetic analysis of currently circulating CDV lineages.

## 1. Introduction

Canine distemper is a disease of dogs and wild carnivores caused by the canine morbillivirus (previously called distemper virus, CDV), belonging to the genus *Morbillivirus*, family Paramyxoviridae [1]. Closely related paramyxoviruses, such as the phocine morbillivirus (phocid distemper virus), rinderpest virus in animals and measles virus in humans are important pathogens [2]. Canine distemper virus is spread worldwide and is associated with highly contagious infection in susceptible species. The main hosts of the virus are carnivores, especially canids [3], in which the infection is clinically manifested with respiratory, gastrointestinal, dermatologic, and neurological disorders [4]. The CDV may also infect animals from the families Ailuridae, Felidae, Hyaenidae, Mephitidae, Mustelidae, Odobenidae, Otariidae, Phocidae, Procyonidae, Ursidae and Viveridae of the order Carnivora and in animals from species of the orders Artiodactyla, Primates, Proboscidea, Rodentia and recently Pilosa [4,5].

The CDV virion contains a 15,690 bp negative-sense single-stranded RNA genome with the hemagglutinin (H) gene as one of the most variable genes [6]. The H gene is therefore a suitable target for molecular epidemiological studies [7,8]. Based on phylogenetic analysis of H gene sequences, CDV is classified into 19 genetic lineages distributed worldwide including America-1 (vaccine strains), America-2 through -5, Europe (also called Europe/South America-1), Europe-Wildlife, Arctic-like, America-1/Europe, Rockborn-like, Asia-1 through -4, India-1/Asia-5, Asia-6, South Africa, and South America-2 and -3 [9,10,11,12].

In Europe, CDV circulates mainly in the wild red fox population. Canine distemper was previously described in northern Italy and in nearby Germany (the federal states of Bavaria, Saxony, and Anhalt) [13,14,15]. In the Czech Republic, only sporadic cases of CDV have been proven in wild mustelids [16]. The last wave of canine distemper attacked the domestic dog population in the Czech Republic between the years 1990 and 1993 [17], however a massive vaccination of domestic dogs prevented the spread of the virus. 

The aims of our study were to assess the prevalence of the CDV in wildlife from the Czech Republic and to use phylogenetic analysis of H gene sequence to determine their evolutionary relationships with the European lineages.

## 2. Results

In total, 74 (18%) of 412 wild animals were positive for the CDV, including 62 (28%) foxes, 4 (10%) stone martens, 2 (20%) pine martens, 2 (2.5%) European badgers, 2 (43%) raccoons and 1 (20%) undetermined marten; animals belonging to the other species were negative (Table 1). There was a statistical difference in positivity among species (*p* < 0.0001). Animal species that had only one representative were excluded from statistical analyses. Scheffé’s method of multiple comparisons showed that at the significance level of 0.05 the pairs (red fox, European badger) and (red fox, European otter) differed. Positive animals were found in 12 of 14 regions with statistical differences among regions (*p* = 0.0057); the highest prevalence 37.5% was in the region Ústí nad Labem. Depending on the year, the prevalence ranged from 41.6% in 2012 to 0% in 2014 with significant statistical differences (*p* = 0.0005).

**Figure 1:**
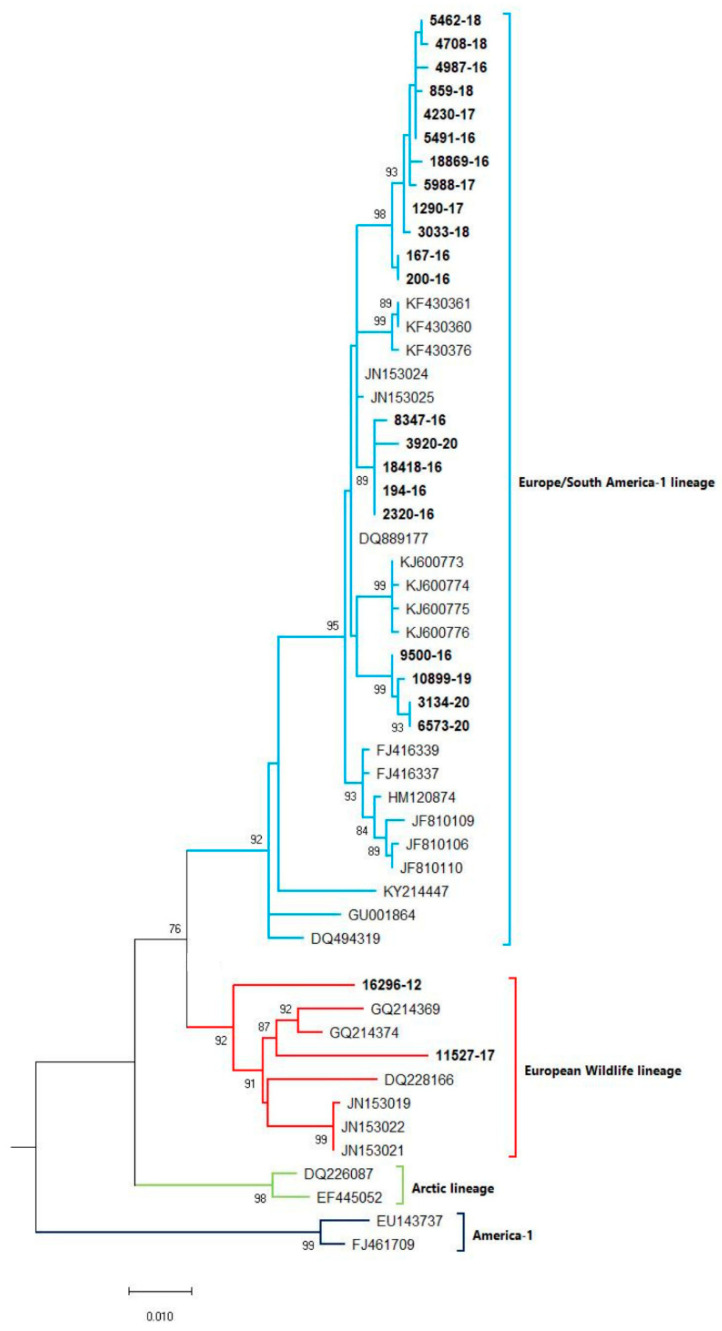
Phylogenetic tree constructed from the Czech CDV (canine distemper virus) variants isolated from wild animals and variants, representing different CDV lineages, available from the NCBI. The sequences from our study are in bold. The evolutionary history was inferred by using the Maximum Likelihood method and Tamura 3-parameter model [18]. The percentage of trees, in which the associated taxa clustered together, is shown next to the branches (bootstrap values 1000 replicants). The tree is drawn to scale, with branch lengths measured in the number of substitutions per site. Evolutionary analyses were conducted in MEGA X [19].

**Figure 2:**
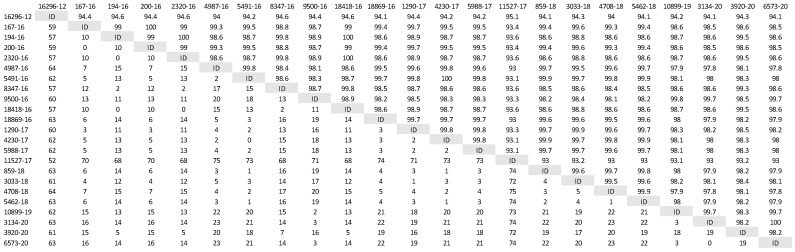
Nucleotide variability of Czech CDV (canine distemper virus) variants isolated from wild animals. The matrix was generated by plotting the CDV variants against each other. The difference in the number of nucleotides is expressed below the diagonal, the nucleotide similarity of the CDV variants in percentages is expressed above the diagonal.

**Figure 3:**
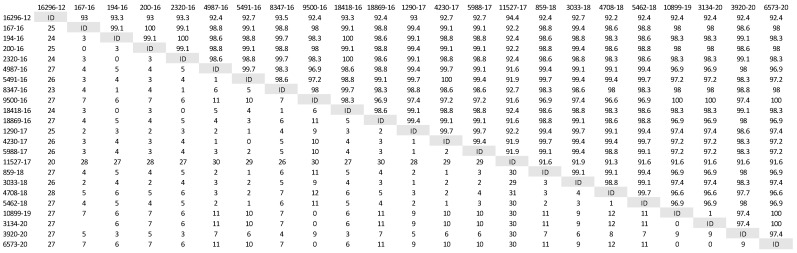
Amino acids variability of Czech CDV (canine distemper virus) variants isolated from wild animals. The matrix was generated by plotting the CDV variants against each other. The difference in the number of amino acids is expressed below the diagonal, the amino acids similarity of the CDV variants in percentages is expressed above the diagonal.

A phylogenetic tree was constructed (Figure 1) based on partial H gene sequences from 23 animals that were positive for CDV (Table 2). Twenty-one variants were assigned to the Europe/South America -1 lineage, suggesting that this lineage is the most prevalent among wild animals in the Czech Republic. Two other variants isolated from the stone marten (11527/17) and the pine marten (16296/12) were characterized as European Wildlife lineage. The genetic relatedness of CDV variants circulating in wildlife population in the Czech Republic with other European variants of CDV is shown in nucleotide (Figure 2) and amino acids variability matrices (Figure 3). The nucleotide sequence differences within 21 Europe/South America -1 lineage reached up to 23 nucleotides, which represents 97.8% similarity. The difference in 23 nucleotides led to the substitution of 12 amino acids, which means that approximately half of the substitutions were non-synonymous. Two variants (no. 9500/16 from a marten and no. 10899/16 from a fox) from the Pilsen region showed high identities of 99.44% and 99.22%, respectively, with the CDV variants isolated from dogs in Hungary during the years 2005–2006 (GenBank accession no. DQ889177). The rest of the 19 variants showed high identities of 98.80–99.73% with the CDV variants isolated from the two foxes in Germany in 2008 (GenBank accession no. JN153024.1 and JN153025.1). Two variants characterized as European Wildlife lineage showed a difference in 52 nucleotides, which represent substitutions in 20 amino acids. The variants no. 16296/12 and 11527/17, isolated from martens, showed a high identity of 97.01% with the CDV variant isolated from the badger from Austria (GenBank accession no. GQ214374.2). These results suggest cross-border transmission of the CDV between the Czech Republic and neighboring countries.

## 3. Discussion

Canine distemper disease was reported in Europe in the second half of the 18th century [20]. In the Czech Republic, the virus was first reported in the 1930s and in the following decades caused the deaths of domestic dogs and farm silver foxes (*Vulpes vulpes*), polar foxes (*Vulpes lagopus*), and American minks (*Neovison vison*) [21]. The last outbreak of canine distemper disease in domestic dogs in the Czech Republic occurred between the years 1990 and 1993. At that time, it affected about 40% of the population of dogs [17]. In the case of foxes, great attention was focused on rabies, that is why there was not any monitoring of the CDV in foxes in the past. There is only one article published on canine distemper in wild mustelids in the Czech Republic [16]. Authors collected 194 samples of brain tissues from wild mustelids in the years 2001–2003 and tested them for the presence of CDV by a direct immunofluorescence test. They detected CDV in stone martens and badgers and observed clinical signs of distemper in three stone marten pup siblings. 

The widespread vaccination of dogs in the first half of the 1990s prevented the spread of CDV and is probably the reason for the eradication of infection in dogs in the Czech Republic [17]. Although several fatal cases of post-vaccination canine distemper were demonstrated in the years 2002–2004, e.g., in domestic ferrets (*Mustela putorius furo*) bred as pets and in the South American coati (*Nasua nasua*) at a zoo (unpublished results), no autochthonous clinical case of distemper in dogs has been published in the last 25 years. Due to the absence of clinical cases in dogs, the occurrence of canine distemper has not been systematically monitored even in red foxes and other wild carnivores in the Czech Republic. 

In our study, we reported CDV predominantly in red foxes, less in both species of martens, badgers, and raccoons, while the virus was not detected in other species. The composition of the species investigated was influenced by the population density of carnivores in the Czech Republic [22]. The red fox has been overpopulated in the Czech Republic for a long time, and it has been regularly hunted and examined, mainly to prevent the spread of rabies (the last case was proven in the Czech Republic in 2002). It appears likely that the red fox plays a major role in the circulation of the CDV in Central Europe. Our results are consistent with previous studies in neighboring Germany [23,24]. The population of stone martens is more numerous than the pine marten population, however both are only rarely hunted and examined in the laboratory. The badger is rarely hunted in the Czech Republic and examined for trichinellosis. The wolf is a rare and protected species in the Czech Republic, but has been increasing in numbers in recent years. Similarly, the European otter is a protected species with an estimated population size of 2500. Raccoons and the American mink are invasive species in the Czech countryside, both of which are occasionally hunted and sporadically examined [22].

In our study, we focused on the long-term collection of samples. In total, 23 partially sequenced variants of the CDV were characterized with respect to their lineages, diversity, the geographical origin, and year of collection. We were limited to the passive monitoring of the CDV in the wild carnivore population (just sick or dead carnivores). However, the results showed that during the period observed (2012–2020), CDV infection showed some dynamism. A total of 47 CDV variants were detected in animals from the Pilsen region, which was also a region where the infection was detected both at the beginning (2012) and at the end (2020) of our study. The Pilsen region is the western region of the Czech Republic, which is directly adjacent to Bavaria (Germany), where the occurrence of the CDV in wild carnivores has been described shortly before [13]. 

It is probable that the situation in the Czech Republic was like that in Germany, with the difference that the CDV in foxes in the Czech Republic escaped the attention of professional observers for a long time. One of the reasons for a such speculation may be the fact that rabies was a more significant and monitored disease until around 2005. The red fox is an adaptable species that has virtually no enemy in the Czech Republic, and therefore, as a successful species, its numbers have increased for several decades. Rabies has been involved to some extent in regulating the numbers of the fox population in the 1990s. After oral vaccination, the fox population recovered, and a gradual increase in the density of fox populations in the Czech Republic continued. The reluctance of hunters to hunt foxes has also contributed to this increase. In a large population of foxes, there is a higher frequency of contacts and therefore easier transmission of infection. The CDV circulated in wild carnivores in varying degrees in different regions of the Czech Republic, as evidenced in mustelids [16]. 

The sequence analysis of 23 CDV variants, randomly selected from the various animal species, regions, and years, showed little genetic variability (heterogeneity) with a predominant lineage Europe/South America-1 in 21 variants. Only two variants were characterized as European Wildlife lineage. The occurrence of these lineages was previously published in wild animals from neighboring countries Austria and Germany [25,26]. The CDV variants selected for sequencing analyses were highly identical to the CDV variants from Germany, Austria, and Hungary [27,28,29]. Similar CDV variants, circulating in wildlife in Central Europe, confirm the cross-border transmission of the CDV between Germany, Austria, and the Czech Republic. With regard to this fact, it cannot be ruled out that, in the future, other CDV lineages may appear in the population of Czech wild carnivores. 

## 4. Materials and Methods

### 4.1. Animals

Samples (brain, lung, and diaphragm) from 412 wild animals representing 10 species were collected in all 14 Czech regions from January 2012 to December 2020 (Table 1). The following species were sampled: 219 red foxes (*Vulpes vulpes*), 79 European badgers (*Meles meles*), 47 European otters (*Lutra lutra*), 40 stone martens (*Martes foina*), 10 pine martens (*Martes martes*), 7 raccoons (*Procyon lotor*), 5 undetermined martens (*Martes* sp.), 2 wolves (*Canis lupus*), 1 European polecat (*Mustela putorius*), 1 free-ranging ferret (*Mustela putorius furo*), and 1 free-ranging American mink (*Neovison vison*). 

A large number of the animals (foxes and badgers) were hunted during the hunting season and their carcasses were sent for examination to the State Veterinary Institute (SVÚ) Prague, SVÚ Olomouc or SVÚ Jihlava. In the case of overpopulated foxes, hunting is legal throughout the year. Foxes showing symptoms such as apathy, loss of shyness or disorientation are monitored for rabies according to the current regulations. The same animals were tested in our study for the presence of CDV. A smaller group of animals including ferret, mink, otter, polecat, wolf and 23 foxes were found dead. Three animals (1 fox, 1 raccoon, and 1 wolf), found in the wild, showed loss of shyness and impaired orientation and were therefore euthanized. 

### 4.2. Molecular Methods

Canine distemper virus (CDV) was detected in the tissues (brain and lung) of animals by real-time RT-PCR. In the case of badgers, CDV was detected in samples of the diaphragm that was collected by hunters and sent to the SVI Prague for detection of *Trichinella* spp. Total nucleic acids were extracted from tissues with the MagNA Pure Compact Nucleic Isolation Kit I (Roche, Basel, Switzerland) and machine TissueLyser II (Qiagen), with input sample volumes of 200 µL and elution volumes of 50 µL. Isolated nucleic acids were stored at −80 °C until they were assayed. For the initial detection of the CDV, a real-time polymerase chain reaction (RT-PCR) targeting N-protein was performed according to the procedure described by Elia et al. [28].

Not all samples positive by RT-PCR were suitable for sequencing. Some of them were weakly positive and thus with a small chance to be successfully sequenced and most of the positive samples were collected in the years 2016 and 2017 from a relatively limited area around Pilsen. That is why positive samples from different species, different regions and years were used together with a representative number of samples from the Pilsen region. Partial Sanger sequencing of the hemagglutinin (H) gene was thus carried out in 23 of 74 CDV variants (Table 2).

First, a reverse transcription and amplification of the H gene, described by Harder et al. [29], was performed using the OneStep RT-PCR kit (Qiagen, Hilden, Germany). The final volume of 50 µL of the master mix contained 0.6 µM of each primer (Table 3) and 5 µL of nucleic acid extract. The reaction was performed under the following conditions: 50 °C 30 min, 95 °C 15 min, 35 cycles (94 °C 30 s, 50 °C 30 s and 68 °C 3 min) and 72 °C 10 min. Then, a nested PCR was carried out using the GoTaq Colorless Master Mix (Promega, Madison, WI, USA) with the final volume of 20 µL containing 0.6 µM of each inner primers and 0.4 mM dNTP. Then 1 µL of the PCR product was added. The characteristics of the two sets of inner primers used in the nested PCR [30,31] are summarized in Table 3. The PCR reaction was performed under the following conditions: 95 °C 5 min, 30 cycles (94 °C 30 s, 50 °C 30 s and 72 °C 1 min) and 72 °C 10 min. The sequencing reactions were prepared using the Big Dye Terminator Cycle Sequencing Kit v3.1 (Thermo Fisher Scientific, MA, USA) and evaluated on the 3130 and 3500 Genetic Analyzers (Life Technologies, Carlsbad, CA, USA). Partial nucleotide sequences 1071 bp long were used for phylogenetic analysis. The partial sequences were aligned to 24 hemagglutinin sequences obtained from the GenBank database using MAFFT [32]. A phylogenetic tree was constructed using the MEGA X software [19]. To reveal nucleotide and amino acid differences in the isolates, the partial sequences were compared in a Sequence Identity Matrix and a Sequence Difference Count Matrix using BioEdit Software [33]. These 23 sequences were deposited into the NCBI GenBank under the accession’s numbers MW828731-MW288753 (Table 2).

### 4.3. Statistical Analysis

The results were statistically analyzed with Pearson’s chi-square test for independence using STATISTICA Cz 12 [34]. We tested the null hypothesis that the occurrence of CDV does not differ among species, regions, or years of sampling. The differences were considered statistically significant if the *p*-value was <0.05. In the case of a statistically significant difference of CDV in some of the variables, Scheffé’s multiple comparison method [34] was subsequently applied. This method was used to identify a statistically significant difference between pairs of species, regions, and years of sampling. Animal species that had only one representative were excluded from statistical analyses.

## Figures and Tables

**Table 1:** Characteristics of 412 wild animals tested for canine distemper virus.

Characteristic	Total Number	Positive (%)	Statistic
**Species**			*p* < 0.0001
American mink (*Neovison vison*).	1	0	
European badger (*Meles meles*)	79	2 (2.5%)	
European otter (*Lutra lutra*)	47	0 (0%)	
European polecat (*Mustela putorius*)	1	0	
Ferret (*Mustela putorius furo*)	1	0	
Pine marten (*Martes martes*)	10	2 (20%)	
Raccoon (*Procyon lotor*)	7	3 (43%)	
Red fox (*Vulpes vulpes*)	219	62 (28%)	
Stone marten (*Martes foina*)	40	4 (10%)	
Wolf (*Canis lupus*)	2	0	
Undetermined marten (*Martes* sp.)	5	1 (20%)	
**Region**			*p* = 0.0057
Central Bohemia	22	5 (22.7%)	
Hradec Králové	4	0	
Karlovy Vary	7	2 (28.5%)	
Liberec	36	2 (5.5%)	
Moravia-Silesia	27	2 (7.4%)	
Olomouc	38	3 (8%)	
Pardubice	2	0	
Pilsen	164	47 (28.6%)	
Prague	9	1 (11%)	
South Bohemia	12	1 (9%)	
South Moravia	39	4 (10%)	
Ústí nad Labem	8	3 (37.5%)	
Vysočina	14	2 (14.3%)	
Zlín	26	2 (7.7%)	
Unknown	4	0	
**Year**			*p* = 0.0005
2012	12	5 (41.6%)	
2013	26	5 (19.2%)	
2014	22	0 (0%)	
2015	26	1 (3.8%)	
2016	161	32 (19.8%)	
2017	63	17 (27%)	
2018	70	5 (7.1%)	
2019	10	1 (10%)	
2020	22	8 (38%)	
**Total**	**412**	**74 (18%)**	

**Table 2:** Characteristics of 23 samples, randomly selected from 74 samples positive for canine distemper virus, used for partial H gene sequencing to characterize CDV lineages circulating among wildlife in the Czech Republic.

Sample No.	Identity No.	Species	Year of Sampling	Region	Locality	Lineage	Access No.
1	167/16	Fox (*Vulpes vulpes*)	2016	South Moravia	Jinačovice	Europe/South America-1	MW828746
2	194/16	Fox (*Vulpes vulpes*)	2016	South Moravia	Vyškov	Europe/South America-1	MW828732
3	200/16	Fox (*Vulpes vulpes*)	2016	South Moravia	Bílovice nad Svitavou	Europe/South America-1	MW828747
4	2320/16	Fox (*Vulpes vulpes*)	2016	Ústí nad Labem	Blatno u Podbořan	Europe/South America-1	MW828733
5	4987/16	Fox (*Vulpes vulpes*)	2016	Pilzen	Bušovice	Europe/South America-1	MW828745
6	5491/16	Fox (*Vulpes vulpes*)	2016	Central Bohemia	Jince	Europe/South America-1	MW828741
7	8347/16	Fox (*Vulpes vulpes*)	2016	Pilsen	Pilsen	Europe/South America-1	MW828734
8	18418/16	Fox (*Vulpes vulpes*)	2016	Liberec	Semily	Europe/South America-1	MW828731
9	18869/16	Fox (*Vulpes vulpes*)	2016	Ústí nad Labem	Teplice	Europe/South America-1	MW828739
10	1290/17	Fox (*Vulpes vulpes*)	2017	Karlovy Vary	Karlovy Vary	Europe/South America-1	MW828736
11	4230/17	Fox (*Vulpes vulpes*)	2017	Central Bohemia	Příbram	Europe/South America-1	MW828740
12	5988/17	Fox (*Vulpes vulpes*)	2017	Central Bohemia	Krašovice	Europe/South America-1	MW828738
13	859/18	Fox (*Vulpes vulpes*)	2018	Pilsen	Nevid	Europe/South America-1	MW828743
14	3033/18	Fox (*Vulpes vulpes*)	2018	Prague	Zbraslav	Europe/South America-1	MW828737
15	4708/18	Fox (*Vulpes vulpes*)	2018	Vysočina	Rančířov	Europe/South America-1	MW828744
16	5462/18	Fox (*Vulpes vulpes*)	2018	Central Bohemia	Benešov	Europe/South America-1	MW828742
17	10899/19	Fox (*Vulpes vulpes*)	2019	Pilsen	Pilsen	Europe/South America-1	MW828748
18	3920/20	Fox (*Vulpes vulpes*)	2020	Pilsen	Blahousty	Europe/South America-1	MW828735
19	16296/12	Pine marten (*Martes martes*)	2012	South Bohemia	Hluboká nad Vltavou	European Wildlife	MW828753
20	11527/17	Stone marten (*Martes foina*)	2017	Pilsen	Pilsen	European Wildlife	MW828752
21	9500/16	Undetermined marten (*Martes* sp.)	2016	Pilsen	Tachov	Europe/South America-1	MW828749
22	3134/20	Raccoon (*Procyon lotor*)	2020	Pilsen	Touškov	Europe/South America-1	MW828750
23	6573/20	Raccoon (*Procyon lotor*)	2020	Pilsen	Křimice	Europe/South America-1	MW828751

**Table 3:** Characteristics of inner primers used in nested PCR used for the amplification of H genes of the canine distemper virus.

Primer	Nucleotide Sequence (5′–3′)	Position ^a^	References
RH3-F	AGGGCTCAGGTACTCCAGC	7059–7077	Harder et al. (1996)
RH4-R	AATGCTAGAGATGGTTTAATT	8975–8995	Harder et al. (1996)
H1F	ATGCTCTCCTACCAAGACAA	7079–7098	An et al. (2008)
H1R	CATGTCATTCAGCCACCGTT	7848–7867	An et al. (2008)
H2F	AATATGCTAACCGCTATCTC	7730–7749	An et al. (2008)
H2RB	TTTGGTTGCACATAGGGTAG	8236–8255	Budaszewski et al. (2014)

^a^ Numerical position on the genome of CDV Onderstepoort strain (GenBank: NC001921).

## Data Availability

The data that support the findings of this study are available on request from the corresponding author. The data are not publicly available due to privacy or ethical restrictions.
